# Heterologous immunity induced by 1^st^ generation COVID-19 vaccines and its role in developing a pan-coronavirus vaccine

**DOI:** 10.3389/fimmu.2022.952229

**Published:** 2022-08-15

**Authors:** Raj S. Patel, Babita Agrawal

**Affiliations:** Department of Surgery, Faculty of Medicine and Dentistry, College of Health Sciences, University of Alberta, Edmonton, AB, Canada

**Keywords:** heterologous immunity, pan-coronavirus vaccine, universal vaccine development, SARS-CoV-2, COVID-19

## Abstract

Severe acute respiratory syndrome virus-2 (SARS-CoV-2), the causative infectious agent of the COVID-19 pandemic, has led to multiple (4-6) waves of infections worldwide during the past two years. The development of vaccines against SARS-CoV-2 has led to successful mass immunizations worldwide, mitigating the worldwide mortality due the pandemic to a great extent. Yet the evolution of new variants highlights a need to develop a universal vaccine which can prevent infections from all virulent SARS-CoV-2. Most of the current first generation COVID-19 vaccines are based on the Spike protein from the original Wuhan-hu-1 virus strain. It is encouraging that they still protect from serious illnesses, hospitalizations and mortality against a number of mutated viral strains, to varying degrees. Understanding the mechanisms by which these vaccines provide heterologous protection against multiple highly mutated variants can reveal strategies to develop a universal vaccine. In addition, many unexposed individuals have been found to harbor T cells that are cross-reactive against SARS-CoV-2 antigens, with a possible protective role. In this review, we will discuss various aspects of natural or vaccine-induced heterologous (cross-reactive) adaptive immunity against SARS-CoV-2 and other coronaviruses, and their role in achieving the concept of a pan-coronavirus vaccine.

## Introduction

Coronaviruses (CoVs) are a diverse family of enveloped, positive sense, single-stranded RNA viruses whose natural hosts are bats and other mammalian species (1). Four genera of coronaviruses exist, *Alphacoronavirus, Betacoronavirus, Gammacoronavirus*, and *Deltacoronavirus*, of which *Alpha-* and *Beta-coronaviruses* are most effective at causing respiratory pathologies and disease in humans (1, 2). Since the beginning of the 21^st^ century, three highly pathogenic strains of beta-coronaviruses have crossed the species barrier to cause zoonotic diseases with high transmissibility and mortalities in humans: severe acute respiratory syndrome virus (SARS-CoV-1) in 2002, Middle Eastern respiratory syndrome virus (MERS-CoV) in 2012, and most recently, severe acute respiratory syndrome virus 2 (SARS-CoV-2) in 2019 (3). The first case of SARS-CoV-2 was reported in Wuhan, China by the Wuhan Municipal Health Commission on 31 December 2019 (4, 5). Due to the rapid spread of the virus across the globe, the World Health Organization (WHO) declared SARS-CoV-2, the causative agent of coronavirus disease 2019 (COVID-19) pandemic in March 2020. Ongoing efforts against SARS-CoV-2 with 1^st^ generation COVID-19 vaccines (Pfizer/BioNTech-BNT162b2, Moderna-mRNA-1273, AstraZeneca-ChAdOx1nCov-18, Johnson & Johnson-Ad26.COV2-S) and the implementation of booster shots have reduced death tolls, hospitalizations, and mitigated severe pathologies associated with COVID-19 disease. Additionally, non-pharmaceutical based interventions such as social distancing, wearing masks, frequent handwashing, and quarantine practices have contributed to slowing the spread of the virus. However, the emergence of increasingly pathogenic and transmissible SARS-CoV-2 variants has prolonged the pandemic. As of April 2022, there are >500,000,000 confirmed cases of COVID-19 and >6,000,000 deaths related to COVID-19 globally (6). As the pandemic continues, protection acquired from natural infection and/or vaccine-induced immunity has been steadily declining (7). Besides specific immunity induced by vaccines, an untapped mechanism of protection, known as heterologous immunity, can provide insight into protective mechanisms of long lasting, cross-variant immunity, and impact the design, development, and implementation of future generations of COVID-19 vaccines.

Heterologous immunity is acquired when the immune response to one pathogen influences the outcome of an infection with unrelated pathogens (8). Heterologous immunity arises from the fundamental characteristics of the adaptive immune system of specificity and memory that allows a quick and efficient response towards previously encountered pathogens or challenges. Immunological memory implies the establishment of lymphocyte populations that can become quickly reactivated upon secondary infection, promoting long-term, sustainable, protective responses. Specificity allows the selection and expansion of epitope-specific T/B cell populations that drive the ability to mount a robust pathogen-specific response against previously encountered pathogens (8–10). Inducing heterologous immunity is centred on promiscuous pathogen-specific T/B cell memory responses that respond against epitopes that may or may not share similarities with other unrelated pathogens. In other words, heterologous immunity supports the idea that pathogen-specific memory responses can mount a robust response against a novel, never-before-seen pathogen *via* a mechanism of cross-reactivity. Heterologous immunity allows the cross-reactivity of immune responses and its range in detecting pathogens that are cross-variant, cross-species, or even cross-kingdom. A study by Youssef et al. identified a Hyrl antigen specific IgM antibody from a fungal infection, *Candida albicans*, that was able to cross-react with two Gram-negative bacteria, *Acinetobacter baumannii* and *Klebsiella pneumoniae* (11). The study found there was structural homology between the hypha-regulated protein (Hyr1) of *C. albicans* and the cell surface protein of *A. baumannii.* Furthermore, the study found that active vaccination with Hyr1p-N protein or passive immunization with the IgM antibody to that protein protects mice from *A. baumannii* infections and prevents severe bacteremia (11). This IgM antibody illustrates a cross-kingdom humoral response that is not only capable of cross-reacting with both a bacterial and fungal protein, but is also functionally protective against *A. baumannii* infections and their pathologies. Additional studies on cross-reactive antibodies and T cell responses among *Plasmodium* species, influenza viruses, and flaviviruses are leading vaccine development in a new direction (12–14). This new approach explores vaccine-induced immunity not only against the targeted pathogen, but also towards closely related, and even distantly pathogens. Therefore, vaccine development efforts also need to emphasize towards a universal vaccine that broadly protects against the targeted pathogens and its cross-reactive counterparts.

## SARS-CoV-2 Origin

**Figure 1:**
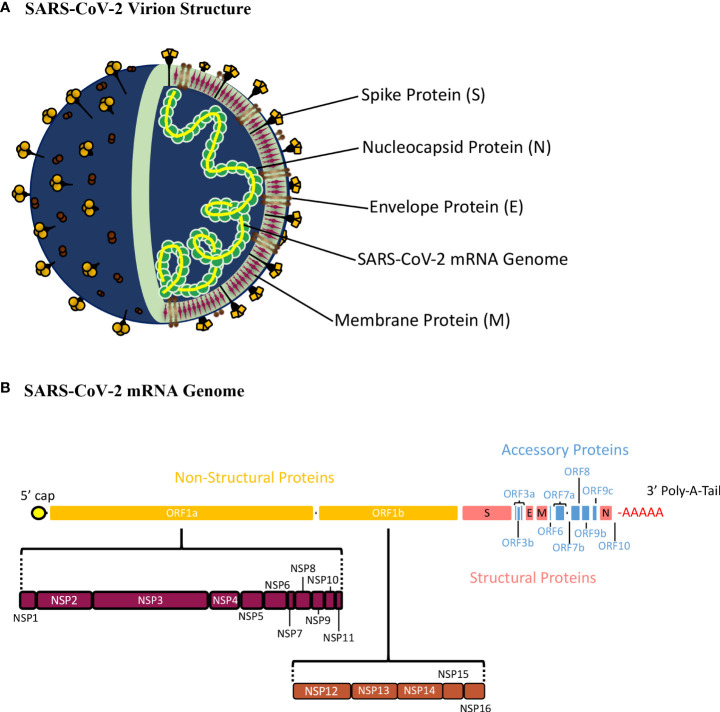
Schematic Presentation of the SARS-CoV-2 Virion Structure and mRNA Genome. **(A)** The SARS-CoV-2 virion consists of the spike (S), nucleocapsid (N), envelope (E), and membrane (E) proteins^22^. The S, M, and E proteins make up the viral envelope, which is the outermost layer of the SARS-CoV-2 virion. The N protein is tightly bound with the SARS-CoV-2 mRNA genome and interacts with M-protein ensuring that proper packaging of the genome occurs within the virion. **(B)** The SARS-CoV-2 genome is a single stranded, positive-sense mRNA genome with a 5’ cap and 3’ poly-A-tail. The full SARS-CoV-2 genome translates 16 non-structural proteins (NSP1-16) from orf1a and orf1b, 9 accessory proteins from orf3a, orf3b, orf6, orf7a, orf7b, orf8, orf9b, orf9c, and orf10, and 4 structural proteins (S, E, M, and N).

SARS-CoV-2 (**Figure 1A**) is a member of the family, *Coronaviridae* and genus, *Betacoronaviruse*—these viruses reside in bat reservoirs and commonly infect humans *via* zoonotic transmission (15). Epidemiological studies looking into sequence and amino-acid similarities between closely-related-SARS-CoV-2 coronaviruses have provided information about the zoonotic origins of SARS-CoV-2. The closest relative of SARS-CoV-2, RaTG13, was discovered from *Rhinolophus affinis* bats in Yunnan Province, China. It had 96.2% sequence similarity to the entire SARS-CoV-2 genome, but the RaTG13 genome lacked two critical regions: the receptor binding domain (RBD) and cleavage-site sequence (15, 16). It was suggested that SARS-CoV-2 and RaTG13 diverged more than 30 years ago into two lineages where RaTG13 was a recombinant virus, and SARS-CoV-2 possessed the ancestral RBD trait that is shared among bat viruses (15, 16). Several reports of coronaviruses from different bat species around the Yunnan Province have shown similarities to SARS-CoV-2. RmYN02, discovered in *Rhinolophus malayanus* bats, shows a 97.2% sequence similarity to the open reading frame (orf)-1a and -1b, which is the largest orf in coronaviruses, extending 21,300 nucleotides (16). RpYN06, found in *Rhinolophus pusillus*, had 94.5% sequence identity to SARS-CoV-2 genome, and it is the closest related genomic backbone to SARS-CoV-2 known to date (17). Like MERS-CoV and SARS-CoV-1, SARS-CoV-2 has originated from bats; furthermore, fecal samples from domestic and wild animals suggested that an intermediate host may have played a role in the SARS-CoV-2 zoonosis (15–17). Reports of closely related coronaviruses from Malaysian pangolins that were smuggled to Guangdong Province in China, exhibited 85.5-92.4% sequence similarity to the SARS-CoV-2 genome, and 97.4% amino-acid similarity to SARS-CoV-2 RBD with identical amino-acid residues in five critical RBD sites (1, 17–21). SARS-CoV-2-related-coronaviruses are known to reside in Malaysian pangolin populations and manifest similar disease outcomes as humans, therefore it is speculated that pangolins could have been an intermediate host for SARS-CoV-2 (17, 18). Collectively, these reports confirm SARS-CoV-2-related coronaviruses exist in wild animals, especially bat species (1, 20). The role of an intermediate host—if there is any—has not been conclusively proven as no direct ancestor of SARS-CoV-2 virus (which is expected to have more than 99% sequence similarity throughout the genome) has been found (21). Currently, the origin of SARS-CoV-2 is suggested to be a result of a cross-species recombination event between a bat and a pangolin coronavirus that subsequently crossed the species barriers to infect humans (1, 15–21).

## SARS-CoV-2 genome and structural/non-structural proteins

SARS-CoV-2 has a 30,000 nucleotide, positive sense, single stranded RNA genome defined by a 5’-cap and 3’-poly-A-tail (**Figure 1B**) (22). The 5’ end of SARS-CoV-2 has a frameshift mechanism between orf1a and orf1b, which allows the synthesis of two polypeptides that are cleaved to produce 16 non-structure proteins (Nsp1-16). These proteins play an important role in the infectious cycle of SARS-CoV-2. Structural proteins such as Spike (S), Nucleocapsid (N), Membrane (M), and Envelope (E) are encoded at the 3’-end, (**Figure 1B**) (22, 23). The SARS-CoV-2 genome is wrapped around the N protein, which is surrounded by a membrane consisting of the M, E and S proteins (21). Also, the 3’ end of the genome encodes nine accessory proteins (orf3a, orf3b, orf6, orf7a, orf7b, orf8, orf9b, orf9c, orf10), which play a role in virulence and host interactions (**Figure 1B**) (24). These accessory proteins are not conserved as well as the structural proteins among coronaviruses, therefore, vaccine and therapeutic efforts focus mainly on the structural proteins of SARS-CoV-2 (7, 25, 26).

In RNA viruses, like SARS-CoV-2, nucleotide changes occur at a higher rate compared to DNA viruses due to its erroneous replication process. This inefficiency alters genomic sequences and facilitates the proliferation of novel variants. The genomic alternations that increase viral fitness, such as enhancing viral entry, replication, immune evasion, and transmission, result in the increased number of the variants that have the desired genomic change (26). In addition to the faulty replication process, the SARS-CoV-2 genome has polymorphic sites concentrated at encoding regions at the 3’ end, leading to more mutations in structural proteins (25, 26). A 2006 study comparing 116 SARS-CoV genomes showed that regions encoding orf10/11, orf3/4, E, M and S proteins are subjected to higher mutation rates compared to other regions of the genome (27). More recently, Mohammad et al. found that emerging SARS-CoV-2 variants have the highest mutation rates in structural proteins in the following order: Spike >Nucleocapsid >Membrane >Envelope proteins (28). Furthermore, they found that certain mutations in structural proteins were associated with advantageous effects on the infection cycle of SARS-CoV-2.

### Spike protein

**Figure 2:**
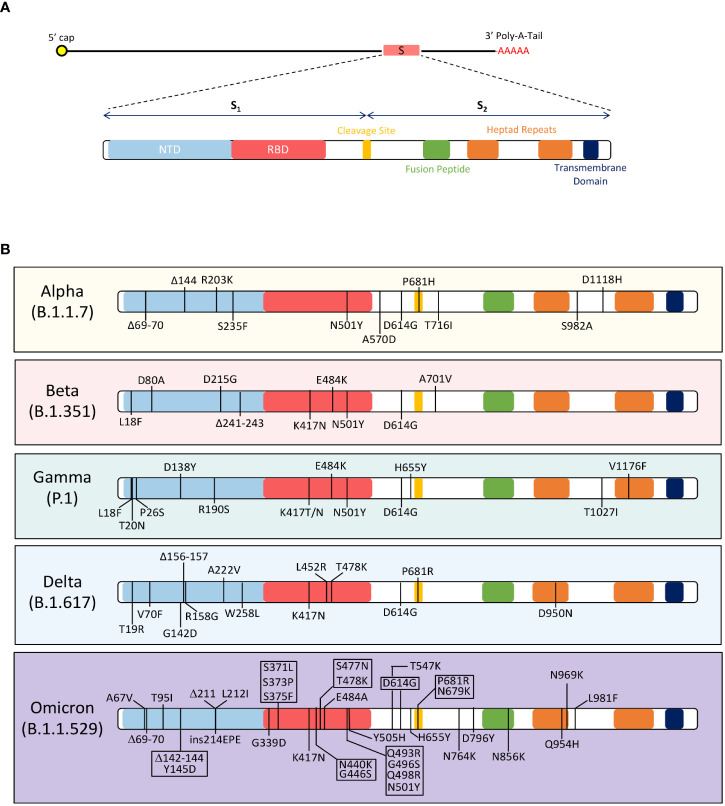
Mutations in the SARS-CoV-2 Spike Protein Among the Alpha (B.1.1.7), Beta (B.1.351), Gamma (P.1), Delta (B.1.617), and Omicron (B.1.1.529) variants of concerns. **(A)** The figure shows the domains of the SARS-CoV-2 spike protein. The spike protein has two subunits, S_1_ and S_2_; these subunits separate at the cleavage site. The S_1_ subunit contains the N-terminus domain (NTD) and receptor-binding domain (RBD). The S_2_ subunit has a fusion peptide, two heptad repeats, and a transmembrane domain. **(B)** The amino acid changes in the spike protein of 5 VOCs are depicted in the figure. The Alpha (B.1.1.7) variant contains these mutations in the spike protein: D614G, ΔH69-V70, ΔY144, R203K, S235F, N501Y, A570D, P681H, T716I, S982A, and D1118H^78,79^. Beta (B.1.351) variant has the following mutations in the spike protein: D80A, D215G, K417N, E484K, N501Y, D614G and A701V^90,91^. Gamma (P.1) variant has L18F, T20N, P26S, D138Y, R190S, K417T/N, E484K, N501Y, D614G, H655Y, T1027I, and V1176F mutations in the spike protein^98 98^. The Delta (B.1.617) variant contains T19R, V70F, G142D, Δ156-157, R158G, A222V, W258L, K417N, L452R, T478K, D614G, P681R, and D950N mutations in the spike protein^105^. The Omicron (B.1.1.529) variant has acquired over 32 mutations in the spike protein, including A67V, Δ69-70, T95I, Δ142-144, Y145D, Δ211, L212I, ins214EPE, G339D, S371L, S373P, S375F, K417N, N440K, G446S, S477N, T478K, E484A, Q493R, G496S, Q498R, N501Y, Y505H, T547K, D614G, H655Y, N679K, P681H, N764K, D796Y, N856K, Q954H, N969K and L981F^105^.

The spike protein is 1273 amino acids long with a molecular weight of 180-200 kDa. It is a type 1 fusion membrane protein, consisting of an extracellular N-terminus domain (NTD), an RBD, a cleavage site, a fusion peptide, two heptad repeats, a transmembrane domain, and a cytosolic C-terminus domain (**Figure 2A**) (29). The extracellular region of the spike protein is highly glycosylated. The S protein has two subunits, S_1_ (14-685 aa residues) and S_2_ (686-1273 aa residues), that dissociate *via* a host furin protease, at its cleavage site (30). In the infection cycle, the S protein is responsible for binding to the host angiotensin-converting enzyme 2 receptor (ACE2), and mediates membrane fusion which induces viral entry (26, 29, 30).

The variability observed in the SARS-CoV-2 variants is dominated by mutations in the RBD region of the S_1_ subunit (26). The RBD region reflects evolutionary relativeness among coronaviruses between humans, cats, and swine species, yet this region is the least conserved (26, 29, 30). Mutations in this region directly impact the binding affinity of the virus towards ACE2 and alters the interaction between the virus and target cell (31). RBD mutations are associated with changes in viral virulence, therefore some variants are more infectious than others. Mutations in the S_2_ subunit have also been hypothesized to influence infectivity of SARS-CoV-2. The S_2_ region relies on the efficiency of furin and serine proteases to access cleavage sites to mediates the fusion of viral and host membranes (30). Therefore, mutations in S_2_ evolve to increase the number of furin and serine cleavage sites and/or increase susceptibility of cleavage events (31). The presence of multiple cleavage site in SARS-CoV-2 has significant implications on infectivity as the absence of these sites lead to a less infectious virus, such as SARS-CoV-1. Mutation in the Spike protein and their effects on virulence are discussed below.

In the highly mutating spike protein, the N501Y mutation, found in the B.1.1.7 (UK), B.1.128.1 (South Africa), B.1.351(Brazil), P.1 (Brazil) and B.1.1.529 (South Africa) variants, increased binding affinity of the RBD to ACE2 receptor (32–34). The amino acid substitution is located within the RBD region of the S_1_ subunit and associated with increased infection rates. This mutation is one of the earliest changes detected in emerging SARS-CoV-2 variants mentioned. Similarly, the K417N substitution, found in the B.1.351 (South Africa, Beta variant), P.1 (Brazil), B.1.617 (India), and B.1.1.529 (South Africa) variants, significantly increased binding affinity of spike to ACE2 (35–38). In addition, K417N contributed to immune evasion by reducing antibody binding affinities against RBD (38). The E484K mutation was found in the B.1.351 (South Africa, Beta variant), P.1 (Brazil), P.2 (Zeta), B.1.525 (Eta), and B.1.526 (Iota) variants (36, 37). The amino acid change alters the electrostatic interactions between antibodies and RBD, decreasing the neutralization capability of SARS-CoV-2 polyclonal serum (39–41). L452R and L18F mutations were associated with similar effects as K417N, in evading the host humoral response against SARS-CoV-2 (39–41).

Additionally, the spike protein has mutations that influence the S protein dissociation and cleaving processes. For example, A570D was first found in the B.1.1.7 (UK) variant that induced a conformational change, along with D614G, that destabilizes the S protein (42, 43). Another mutation, S982A, found in the B.1.1.7(UK) and P.3 (Japan/Philippines) variants prevented the formation of hydrogen bonds between S_1_ and S_2_ subunits, increasing the likeliness of these subunits to dissociate (44). Mutations directly involved in the furin cleaving process were characterized by P681H and D614G changes and associated with higher infectivity and replication rates of SARS-CoV-2 (43, 45). All in all, the spike is a highly variable protein that significantly impacts SARS-CoV-2’s ability to infect target cells (28). The 1^st^ generation COVID-19 vaccines are spike-based which have been effective in mitigating COVID-19 associated pathologies, hospitalizations, and deaths. Generally, spike-based immunity has been defined as neutralizing as it prevents the virus from entering targeted host cells. Evidently, during the pandemic, the spike protein has acquired many mutations (**Figure 2B**), and this results in the decline of vaccine-induced immunity. For future vaccines and longer-lasting immunity, it may be important to consider other proteins and antigen targets that are conserved and have lower tendencies to acquire mutations.

### Envelope protein

The envelope protein is ~75 amino acid residues long with a molecular weight of 8-12 KDa, consisting of a hydrophilic extracellular N-terminus, followed by a hydrophobic transmembrane domain, and cytoplasmic C-terminus (46). The envelope protein is responsible for the activation of inflammasomes, and the budding and release of the viral progeny (47). In addition to these functions, the envelope protein forms a pentameric ion channel with low ion selectivity, called a viroporin (47, 48). Many animal studies have suggested that the viroporin plays an important role in virulence and have shown that its ion channel activity is directly proportional to SARS-CoV-2 pathogenesis (47–49). In mouse models, knocking out the envelope gene was found to have no impact on SARS-CoV-2 viral replication, but it reduced edema (47, 50). Many animal studies, where the virus lacks viroporin activity, show reduced IL-1β, TNF-α and IL-6 production in the lungs (47). It is suggested that viroporins and their ion channel activity are linked to the development of cytokine storm and acute respiratory distress syndrome (ARDS).

The envelope protein is a highly conserved protein among various members of coronaviruses, therefore, very few mutations are observed in the E protein (46). Surveying all the SARS-CoV-2 variants, B.1.351 (South Africa, Beta variant) had a P71L mutation in the envelope protein that associated with disease severity and death rate (51). The low variability of the envelope protein makes it an attractive target as a vaccine and therapeutic target, as many circulating viral variants would be vulnerable. Also, the ion channel activity associated with pathogenesis makes the protein immunogenic and potentially capable of inducing a robust immune response. A conserved, immunogenic protein, like the E protein, can be a potential target for immunotherapeutics or vaccine candidates.

### Membrane protein

The membrane protein is a glycoprotein, 222 amino acid residues long, and the most translated protein (22, 52). The membrane protein is a self-formulating protein that interacts with all structural proteins (S, E and N) to form virus-like particles (VLP) (46, 53). The membrane protein is involved in stabilizing the interaction between genomic mRNA and the N-protein complex to incorporate the SARS-CoV-2 genome into the virion (46). Protein-protein interactions between M and other structural proteins allow the trafficking of these proteins for assembly and release. Lastly, the membrane protein is involved in the budding and release of the newly synthesized virions.

Evolutionarily, the M protein has been conserved across *Beta-coronaviruses* providing a common viral structural protein for all viruses within the genus (54). Genomic sequencing reports have shown more than 97% homology between M sequences of SARS-CoV-2 variants sampled (55, 56). Troyano-Hernáez et al. surveyed 103,419 SARS-CoV-2 M sequences, and reported 99.99% conservation, with only 291aa changes found (55). Out of 291 aa changes, only two mutations were presented in significantly higher frequencies: D3G (0.7%, in 724 sequences) and T175M (1%, in 1026 sequences). The D3G mutation first emerged out of Africa and South Africa, and the T175M was discovered in Europe. Another study surveyed 5677 GenBank records and found that 5557 sequences had no mutations or synonymous mutations, while the other 120 sequences had non-synonymous mutations in the M protein (56). Furthermore, the study found 10 mutations (C64F, A69S, A69V, V70F, N113B, R158L, V170I, D190N, D209Y and S214I) in the M protein that are predicted to change secondary protein structure. All in all, the M protein has evolved and incorporated mutations, but it is largely conserved across *Betacoronaviruses* and SARS-CoV-2 variants. With the M protein being involved in integral parts of the infection cycle, such as virus assemble, protein trafficking, and dictating protein-protein interaction of SARS-CoV-2 structural proteins, any major changes can result in detrimental effects on the overall fitness of the virus. Vaccine and immunotherapeutic targeting the SARS-CoV-2 membrane protein can potentially induce immune responses that can cross-react with various SARS-CoV-2 variants and other *Betacoronaviruses* because of the high sequence homology in the M protein.

### Nucleocapsid protein

The nucleocapsid protein is 419 aa residues long (57). It consists of a N-(NTD) and C-terminus domain (CTD) connected by a serine/arginine linker region (LKR). The N protein helps package the viral RNA genome and ensures proper replication and viral assembly processes take place. The LKR allows the N protein to oligomerize with other N-proteins and exposes the RNA-binding domains, located within the NTD/CTD, to bind to the viral genome (58–60). In this process, serine/arginine amino acids will stabilize the interaction between the N protein and RNA genome forming a stable ribonucleoprotein complex, called a capsid (58). The role of the viral capsid is to protect the viral genome from environmental conditions and host immune responses. It also plays an important role in delivery of the genetic contents into targeted cells.

Among coronaviruses, the N protein has been largely conserved, except for certain regions. There is 90% sequence identity between the SARS-CoV-2 and SARS-CoV-1 N proteins (61). Within the N protein sequence, the LKR (182-247 aa residues) accounts for the majority of the mutations in the SARS-CoV-2 variants. These mutations are located around multiple, highly phosphorylated sites that influence N protein thermodynamic properties and have a major impact on the viral life cycle. The R203K mutation is located in the LKR region and was first discovered in the B.1.1.7 (UK) and P.1 (Brazil) variants (62). This mutation coincides with G204R and has been found to increase overall expression levels of the N protein and various sub-genomic RNA transcripts (63). Structurally, the 203/204 mutations increase entropy and intrinsic disorder causing changes to the N protein oligomerization process (62, 63). Next, the T205I mutation was first observed in the B.1.351(South Africa) variant (63). It is a highly phosphorylated site within the LKR. This mutation alters the activation of the N protein resulting in decreased fitness of the virus, however the T205I mutation was reported 43% of the time in B.1.351 variants. Lastly, the S235F mutation was reported in the B.1.1.7 (UK) variant (63). The S235F mutation is unique in that it is associated with immune evasion. The mutation alters a highly targeted B cell epitope, thereby decreasing the specificity of certain antibodies to mount a response against SARS-CoV-2 N protein (63). Immunity against the SARS-CoV-2 N protein is governed by CD4^+^ T cells, and they have been shown to cross-react with other endemic coronaviruses (28).

## Emergence of SARS-CoV-2 variants

SARS-CoV-2 is an evolving virus that manifests a vast number of mutations. Mutations can be induced by errors in replication, however to explain the accelerated proliferation of SARS-CoV-2 variants, two other sources of mutations need to acknowledge: host RNA editing and modification mechanisms, and genetic recombination events (64). RNA editing and modification enzymes, including apolipoprotein B mRNA editing catalytic polypeptide-like enzyme (APOBEC) and adenosine RNA specific 1 enzyme (ADAR1), are host innate anti-viral mechanisms designed to detect viral RNA and alter nucleotide sequences (64, 65). These RNA enzymes can induce cytosine-to-uracil and adenosine-to-inosine nucleotide substitutions in the genomic sequence resulting in translated viral proteins that are dysfunctional (66). Next, phylogenetic studies have revealed that SARS-CoV-2 variants are most likely to have emerged because of recombination events (67–69). Evidence shows that multiple SARS-CoV-2 variants can infect a host, making it more likely that variants can exchange genetic material (70). A UK study found that circulating viruses, from late 2020 to early 2021, were recombinant viruses derived from the B.1.1.7 variant (71). Genomic analysis confirmed the recombination events occurred between ancestral B.1.1.7 and non-B.1.1.7 circulating virus as the recombinant viral genomes acquired single-nucleotide polymorphisms and deletions that define the B.1.1.7 lineage and other non-B.1.1.7 lineage mutations and variations (71). These modifications allow the virus to accumulate mutations and potentially give rise to novel variants. However, viruses have evolved to exploit these RNA enzymes to reach higher evolutionary potential by accumulating and selecting for advantageous mutations.

The WHO continuously monitors the evolution of SARS-CoV-2 and assesses the risk associated with emerging SARS-CoV-2 variants to global public health (72). SARS-CoV-2 variants are categorized into two groups: variants of interest (VOI) and variants of concern (VOC), based on its threat to public health. VOIs are associated with genetic mutations that could affect transmission, diagnosis, treatment and vaccine escape, but cause unique outbreak clusters of cases and have limited prevalence or expansion in many countries. However, VOCs are a major concern for public health as variants are associated with higher transmissibility and expansion in population, increased disease severity, hospitalizations and deaths, and significantly reduced vaccine efficacy rates and/or diagnostic detection failures. All variants of SARS-CoV-2 can be tracked back to the original wild-type strain, Wuhan-Hu-1, isolated in Wuhan in December 2019 that initiated the COVID-19 pandemic. The VOCs that emerged from the Wuhan-Hu-1 SARS-CoV-2 strain are discussed below (**Figure 2B**).

### B.1.1.7 (Alpha) lineage

The B.1.1.7 variant was first discovered in the UK on September 20, 2020 (73). The virus consisted of several novel spike mutations, including 17 non-synonymous, six synonymous, and two deletions: ΔH69-V70, ΔY144, R203K, S235F, N501Y, A570D, D614G, P681H, T716I, S982A, and D1118H (**Figure 2B**) (34, 73). Three of the mutations in the S protein that were reported to be concerning due to an increase in viral transmission, pathogenicity, infectivity, and decreased susceptibility to neutralizing antibodies (34, 72–77). These mutations included the previously discussed N501Y and P681H mutations, and the ΔH69-V70 deletion (3, 34). The ΔH69-V70 deletion, located in the RBD, led to a conformational change in the S protein that evaded the host immune response (78). Additionally, a peer-reviewed study reported a 75% increase in transmissibility of the B.1.1.7 variant compared to the Wuhan-Hu-1 strain (74). Within three months, the B.1.1.7 variant was the predominant circulating strain in the UK and spreading across 114 countries (74–77). In terms of diagnosis, Public Health England reported that the B.1.1.7 mutations in the S gene alluded the RT-PCR detection assay—which amplifies the S gene to detect COVID-19 positivity in sick patients (79). In March 2021, Ontario’s COVID-19 Science Advisory Committee reported that 67% of COVID-19 cases were due to the B.1.1.7 variant (80, 81). These cases were associated with a 63% increase in hospitalization risk, 103% increase in intensive care admissions related to COVID-19 and 56% increase in mortalities (75, 81). With increased infectivity and transmissibility, the B.1.1.7 variant was reported to target younger age groups in Canada, between ages 20-30 y and up to 59 y (82).

### B.1.351 (Beta) lineage

The B.1.351 emerged from South Africa in October 2020 (83). From October 2020 to February 2021, the variant had spread to over 40 countries, including South Africa, Philippines, USA, Canada, and several EU countries. This lineage is defined by multiple mutations in Spike, including L18F, D80A, D215G, Δ241-243, K417N, E484K, N501Y, D614G and A701V (**Figure 2B**) (83, 84). Other mutations in N, E and orf1a proteins, include T205I, P71L and K1655N substitutions, respectively (83). In these B.1.351 variants, E484K and K417N mutations were concerning as they compromised neutralizing responses established from natural SARS-CoV-2 infections and/or vaccine-induced immune responses (85). Many studies reported that E484K, K417N and N501Y mutations can escape vaccine induced neutralizing antibody responses, as much as 3-5 folds (86, 87). In addition to immune evasion, the B.1.351 variant was 50% more infectious compared to its ancestor, as it dominated the second wave in South Africa, infecting more than 1.3 million people and causing 37,000 deaths (88, 89). Surges in B.1.351 cases demonstrated that the variant was more transmissible and infectious, but no evidence of increased severity was reported. In a molecular study, it was determined that sensitivity of the RT-PCR diagnostic test was not affected by the B.1.351 variant (90).

### P.1 (Gamma) variant

The P.1 lineage emerged out of Brazil in December 2020. It incorporated 17 non-synonymous mutations, 3 deletions, 4 synonymous mutations and one four-nucleotide insertion. Mutations located in the S protein were L18F, T20N, P26S, D138Y, R190S, K417T, E484K, N501Y, D614G, H655Y, T1027I, and V1176F (**Figure 2B**) (91). Other mutations included, P80R in the N protein, S1188L and K1795Q in orf1ab, G174C in orf3a, E92K in orf8, two-nucleotide deletions at positions 11288/9, deletion in the nsp6 protein (S106del, G107del, and F108del), and a four-nucleotide insertion at the orf8/N intergenic region (ins28263) (92–97). The triple mutation in Spike protein of K417T, E484K, and N501Y retained its function of evading both vaccine-induced and natural-infection acquired immunity. In its entirety, the spike mutations in the P.1 lineage associated with higher affinity for ACE2 receptor compared to non-P.1 lineages. This potentially led to a 2.5-fold increase in transmission compared to previously circulating variants and had a reinfection probability of 6.4% (93). From December 2020 to January 2021, the P.1 variant became the dominant variant in Manaus, Brazil as 85% of genotyped samples were found to be P.1 positive (93–95). The WHO announced the P.1 lineage as a VOC, due to its increased transmissibility and its propensity to re-infect individuals.

### B.1.617 (Delta) lineage

The B.1.617 lineage emerged out of India in December 2021 (98). The S protein lineage-defining mutations include, T19R, V70F, G142D, Δ156-157, R158G, A222V, W258L, K417N, L452R, T478K, D614G, P681R, and D950N (**Figure 2B**) (98). Researchers identified two potential mutations, L452R and E484Q, previously reported in B.1.1.7/B.1.351 and B.1.427/B.1.429 variants, respectively, as the reason for the exponential infectivity and immune evasion ability of B.1.617 variants (75, 99). Many viral fitness parameters were enhanced by the acquired mutations in the B.1.617 (Delta) variant. The onset of symptoms after exposure shortened from an average of 16.7 days to 10.3 days in unvaccinated individuals, indicating an enhanced replication ability of the B.1.617 lineage (99–102). A study in India reported that B.1.617 infections were associated with higher viral load and shedding as B.1.617 infections (n=47; Mean Ct: 16.5 cycles) has lower cycle thresholds compared to non-B.1.617 infections (n=22; Mean Ct: 19 cycles) (103). According to the Centre for Disease Control and Prevention (CDC), the reproduction number (R_0_) was between 5-9 for the lineage; in other words, the variant could be more transmissible than other viruses, such as Smallpox, Influenza, MERS, and SARS (102, 104). Moreover, the P681R mutation, previously identified in B.1.1.7 variants, was reported to increase fusion activity *via* enhanced furin cleaving which could possibly be contributing to the increased infectivity and transmissibility seen in the B.1.617 variant (27–32). In relation to disease severity, one study showed infection with the B.1.617 variant in hamsters caused a higher viral load in the lungs, substantial lung lesions and weight loss, compared to other B.1 variants (105). The B.1.617 variants doubled the risk of hospitalizations compared to the B.1.1.7 variant, and patients infected with B.1.617 had severe disease outcomes and higher in-hospital morality rates (106, 107). Vaccine efficacy and neutralizing responses had significant fold-decreases with the introduction of the B.1.617 variant. The B.1.617 variant was the most devastating SARS-CoV-2 variant ever seen, in terms of its ability to replicate, transmit, infect, and inflict pathologies as well as mortality (107–109).

### B.1.1.529 (Omicron) lineage

The first reports of B.1.1.529 emerged from South Africa, in November 2021 (98). Several studies confirmed that the B.1.1.529 spike protein had 32 mutations, including amino acid substitutions, insertions, and deletions (A67V, Δ69-70, T95I, Δ142-144, Y145D, Δ211, L212I, ins214EPE, G339D, S371L, S373P, S375F, K417N, N440K, G446S, S477N, T478K, E484A, Q493R, G496S, Q498R, N501Y, Y505H, T547K, D614G, H655Y, N679K, P681H, N764K, D796Y, N856K, Q954H, N969K and L981F) (**Figure 2B**) (98). The overwhelming number of mutations were predicted to result in the most virulent, infectious, transmissive, and pathogenic virus yet. However, the acquisition of these mutations reduced disease severity clinically (110). Hui et al. found that the B.1.1.529 variants had a lower replication rate compared to other SARS-CoV-2 variants (110). The paper suggests the reduced replication efficiency as a potential explanation for reduced disease severity in patients infected with B.1.1.529. In South Africa, hospital admissions lowered by 29%, compared to the first wave in 2020, highlighting less severe disease outcomes associated with the B.1.1.529 variant (111). The U.S. Food and Drug Administration and Public Health Ontario reported that Antigen Diagnostic Tests were less sensitive in detecting the B.1.1.529 variant as more false negative outcomes were registered (112, 113). Further analysis revealed a 9-nucleotide deletion resulting in failure to detect the SARS-CoV-2 B.1.1.529 variant (113, 114). According to Ontario’s Risk Assessment report, the B.1.1.529 variant dominated COVID cases with numbers increasing from 838 to 14449 within a two-week period in December 2021 (112). In South Africa, B.1.1.529 case numbers increased by 2.5 folds within November 2021 (75). The B.1.1.529 variants and its closely related sub-variants, BA.1 and BA.2, were reported to have alarming rates of transmission, making it a major concern for public health (115, 116).

## 1st generation COVID-19 vaccines-induced immunity against SARS-CoV-2 VOCs

The timely development of the current 1^st^ generation COVID-19 vaccines, including Pfizer/BioNTech (BNT162b2), Moderna (mRNA-1273), AstraZeneca (ChAdOx1nCov-19), and Johnson and Johnson (Ad26.COV2-S), have significantly reduced case numbers, hospitalizations, and deaths related to COVID-19 (117). These vaccines have a common spike-based design from the wild-type, Wuhan-Hu-1 isolate, which is intended to elicit neutralizing antibody responses against the SARS-CoV-2 spike protein (117, 118). Antibody responses against the spike protein has been associated with protection against COVID-19 infections. However, SARS-CoV-2 variants, from alpha to omicron, are consistently mutating the S protein, resulting in the reduction of vaccine efficacy rates of these vaccines as the neutralizing antibodies are unable to recognize novel S protein alterations. Therefore, immunity against spike can be neutralizing, but cannot be sustained for long-term protection against heterologous SARS-CoV-2 S proteins and future variants. Interestingly, all four vaccines have a similar antigenic composition, but the immunity generated from these vaccines have different capacities of inducing heterologous immunity. The 1^st^ generation vaccines are an example of how immunizations with the wild-type SARS-CoV-2 S protein elicits varying degrees of protection against novel variants of SARS-CoV-2, which have different compositions of spike mutations compared to the Wuhan-Hu-1 strain (**Table 1**).

**Table 1:** Summary of reported vaccine effectiveness, vaccine efficacies against hospitalizations/COVID-19 disease, and antibody neutralization of 1^st^ generation COVID-19 vaccine against five variants of concerns: alpha (B.1.1.7), beta (B.1.351), gamma (P.1), delta (B.1.617), and omicron (B.1.1.529) variants.

		Alpha (B.1.1.7) Variant	Beta (B.1.351) Variant		Gamma (P.1) Variant		Delta (B.1.617) Variant	Omicron (B.1.1.529) Variant
AstraZeneca (ChAdOx1nCov-19)	Vaccine Effectiveness	70.4%^127-129^	–		77.9%^130^		67.0%^131^	34-37%^133,134^
	Vaccine Efficacy Against Hospitalization/COVID-19 Disease	Protective (100%)^128,129^	No Protection^126^		Protective (87.6%)^130^		Protective^131^	–
	Antibody Neutralization	Preserved^126^	No Neutralization Capacity^81,126^		–		9-Folds Reduction^133,134^	–
Johnson and Johnson (Ad26.COV2-S)	Vaccine Effectiveness	–	–		–		76.0%^145^	–
	Vaccine Efficacy Against Hospitalization/COVID-19 Disease	–	–		73.1-81.7%^138^		81.0%^145^	63.0%^156^
	Antibody Neutralization	Preserved^141-144^	3.6-Fold Reduction^140^		3.4-Fold Reduction^140^		1.6-Fold Reduction	No Neutralization Capacity^146-149^
Moderna (mRNA-1273)	Vaccine Effectiveness	100%^161,162^	96.4%^161,162^		–		86.7%^161,162^	44.0%^168,169^
	Vaccine Efficacy Against Hospitalization/COVID-19 Disease	Protective^160-163^	Protective^160-163^		Protective^160-163^		Protective^160-163^	Reduced Protection^160-163^
	Antibody Neutralization	1.2-Fold Reduction^171^	8.4-Fold Reduction^171^		3.2-Fold Reduction^171^		3.3-Fold Reduction^171^	–
Pfizer/BioNTech (BNT162b2)	Vaccine Effectiveness	93.7%^173-176^	75.0%^173-176^		–		88.0%^173-176^	70%^173-176^
	Vaccine Efficacy Against Hospitalization/COVID-19 Disease	95% Effectiveness at Protecting Against COVID-19 Disease^173^						
	Antibody Neutralization	2.6-Fold Reduction^177,178^		4.9-Fold Reduction^177,178^		–	5.8-Fold Reduction^177,178^	41-Fold Reduction^177,178^

The dash (-) is placed where data was not available for the defined parameter.

### Oxford-AstraZeneca (ChAdOx1nCov-19) vaccine

The Oxford-AstraZeneca COVID-19 vaccine (ChAdOx1nCov-19) is a non-replicating viral vector vaccine (119). It utilizes a chimpanzee adenovirus vector encoding the wild-type SARS-CoV-2 S protein. The two-dose regimen of ChAdOx1nCov-19 induces a robust immune response composed of IgG_1_/IgG_3_ antibodies and T cell responses directed at Wuhan-Hu-1 spike protein. It also activates a T_H_1 response *via* CD4^+^ T cell secretion of IFN-γ/TNF-α (117, 119). The immunity induced by ChAdOx1nCov-19 has been targeted towards the RBD as both antibody and T cells epitopes are specific for that region of spike. ChAdOx1nCov-19 vaccination trials conducted in the UK (11 636 participants) reported an overall vaccine efficacy rate of 70.4% (95.8% CI, 54.8-80.6) (120–122). Furthermore, 21 days after the 1^st^ dose, there were zero hospitalizations reported within the vaccinated group compared to 15 hospitalizations in the control group (121, 122). Their analysis showed that the two-dose regimen of ChAdOx1nCov-19 requires more than 14 days after the 2^nd^ dose to effectively protect against COVID-19 disease (122). ELISA-based virus neutralization assays revealed that the antibody response induced by ChAdOx1nCov-19 has preserved its effectiveness in protecting against severe disease outcomes of B.1.1.7 (Alpha) variants, however a marginal reduction was observed (119).

Following the B.1.1.7 (Alpha) wave, the B.1.351 (Beta) variant dominated the COVID-19 landscape in many countries around the world. Vaccine efficacy studies on ChAdOx1nCov-19 reported ineffectiveness against B.1.351 as antibody neutralizing ability was reduced against the D614G spike mutation (75, 119). Thus, the humoral response against SARS-CoV-2 is highly compromised by viral mutation. A single amino acid change has the ability make an antibody response ineffective as the study reported vaccine efficacies dropped to 10.4% against mild-to-moderate disease caused by B.1.351 variants. These results caused several countries to halt rollout plans for the AstraZeneca vaccine (75). A more positive experience occurred in Brazil where a two-dose regimen of AstraZeneca was implemented during the epidemic with the P.1 (Gamma) variant. Reports out of Brazil indicated that one dose of ChAdOx1nCov-19 had mitigating effects against COVID-19 as hospitalizations and deaths decreased by 55.1% (95% CI, 46.6-62.2) and 61.8% (95% CI, 48.9-71.4), respectively (123). Moreover, after two doses of ChAdOx1nCov-19, administered three months apart, the effectiveness against the P.1 variant was 77.9%, and hospitalizations and deaths were reduced to 87.6% (95% CI, 78.2-92.9) and 93.6% (95% CI, 81.9-97.7), respectively (123). Similarly, the AstraZeneca vaccine has shown effectiveness in reducing hospitalizations and risk of COVID infections against the B.1.617 (Delta) variant (75, 124). However, the vaccine effectiveness of ChAdOx1nCov-19 against B.1.617 is diminishing when compared to the B.1.1.7 (Alpha) variant (124–126). The effectiveness of the two-dose regimen was 74.5% (95% CI, 68.4 to 79.4) against the B.1.1.7 variant and 67.0% (95% CI, 61.3 to 71.8) against the B.1.617 variant (124). Compared to the Wuhan-Hu-1 SARS-CoV-2 strain, sera from AstraZeneca-vaccinated individuals have reduced neutralizing ability against B.1.617 variant by 9-fold (125). The overall trend observed as novel SARS-CoV-2 variants emerge is that the AstraZeneca vaccine is becoming less effective. Furthermore, vaccine effectiveness has significantly been compromised with the emergence of the B.1.1.529 (Omicron) variant (75). Andrews et al. found there was no protection after 15 weeks of completing the two-dose regimen of ChAdOx1nCov-19, and vaccine efficacy dropped to 34-37%, against the B.1.1.529 variant (127). The study also showed that AstraZeneca recipients, with a Pfizer (BNT162b2) booster restored effectiveness against the B.1.1.529 infections to 92.6% (127). Taken together, it appears that there are cross-reactive mechanisms induced by ChAdOx1nCov-19 that promote cross-protection against the B.1.1.7 and P.1 variants. Cross-reactive mechanisms are critical and desirable for heterologous immunity as these mechanisms will orchestrate and dictate the protective ability against SARS-CoV-2 variants and heterologous pathogens (117). The ChAdOx1nCov-19 vaccine is effective at inducing heterologous immunity as immune responses are providing a certain degree of effectiveness against B.1.1.7 (Alpha), P.1 (Gamma), and even B.1.617 (Delta) variants (117–127). In contrast, it is evident that ChAdOx1nCov-19 induced immunity was not cross-protective towards the B.1.1.529 (Omicron) variant (117–127).

### Johnson & Johnson (Ad26.COV2-S) vaccine

The Johnson & Johnson (J&J) vaccine is a non-replicating viral vector vaccine composed of a human adenovirus type 26 incorporating a full-length wild type S protein (128). J&J vaccine, Ad26.CoV2-S, is a single-dose regimen that generates a robust antibody response targeting the spike protein and RBD (129). Spike and RBD-specific IgA_1_, IgA_2_, IgG_1_, IgG_2_, IgG_3_, IgG_4_, and IgM antibodies were generated from Ad26.CoV2-S and found to cross-react with SARS-CoV-1 S protein and other human coronaviruses such as CoV-229E, CoV-HKU1, CoV-NL63 and CoV-OC43, *in vitro* (129). The CoV1001 Phase I-IIa clinical trial reported humoral and cellular responses against B.1.1.7 (Alpha), B.1.351 (Beta), and P.1 (Gamma) variants in Ad26.CoV2-S vaccinated individuals (129–135). Regarding the B.1.1.7 (Alpha) variant, sera from Ad26.CoV2-S recipients were tested for their neutralizing activity with a pseudovirus inhibition neutralization assay, reporting that Ad26.CoV2-S has significant neutralization ability against B.1.1.7 (132–135). They found that antibody-dependent cellular phagocytosis, complement pathways and natural killer cells were activated against B.1.351 infections (131). In contrast, pseudovirus neutralization assay results indicated that the effectiveness of neutralizing antibody responses were lowered 5.0-fold and 3.3-fold against B.1.351 and P.1 variants, respectively (131). Although protection against infection was reduced significantly, a vaccine safety study conducted in South Africa reported a single dose of Ad26.CoV2-S protected against severe-to-critical disease outcomes, reducing COVID-19-related hospitalizations and deaths (130). The study reported a vaccine efficacy of 73.1% and 81.7% against moderate-to-critical COVID-19, on days 14 and 28 post-vaccination, respectively (130). The antibody response induced by Ad26.CoV2-S has cross-variant capabilities that recognized the B.1.1.7 (Alpha), B.1.351 (Beta), and P.1 (Gamma) variants, and clinically exhibited protection against VOCs, despite waning neutralization ability. Ad26.CoV2-S vaccination induced multiple antibody isotypes, including IgA, IgG, and IgM, which allows antibody protection at many sites of the body, including mucosal barriers, blood, lymphatic fluids, and other extracellular fluids. Inducing a diversified antibody response may be linked to better protective outcomes against SARS-CoV-2 variants. Collectively, an IgA/IgG/IgM response increases the coverage of the humoral immunity to multiple locations around the body and broadens the function of the antibody response, compared to an antibody response mediated by a single antibody isotype as seen with ChAdOx1nCov-19 (131). Notably, IgA antibodies play an important role in fighting infections in the respiratory tract and serve to defend against cell entry of respiratory viruses. Specifically with SARS-CoV-2 infections, a robust IgA response can potentially have neutralizing effects as the virus can be eliminated before reaching the respiratory epithelia and binding to ACE2. Early indicators of a robust IgA response and increased sampling of antibodies at the mucosal barriers may serve as a strong indicator of protection against SARS-CoV-2 infections. In addition to a diversified humoral response, non-neutralizing CD4^+^/CD8^+^ T effector and central memory cells were found to response to SARS-CoV-2 variants in vaccinated individuals. These memory populations may contribute to the protection against disease severity, but the role of these populations is still unclear (131).

Following the B.1.351 and P.1 variants, the B.1.617 (Delta) variant dominated the United States. A cohort study with 422 034 Ad26.CoV2-S vaccinated individuals reported vaccine efficacy rates of 76% against B.1.617 (Delta) variant infections, and 81% against COVID-19 related hospitalizations (136). These vaccine efficacies were maintained for 180 days after vaccination. Additionally, a neutralization study found that sera from Ad26.CoV2-S recipients was reduced 1.6-fold against the B.1.617 variant, while the most noteworthy reduction was observed against B.1.351(3.6-fold) and P.1(3.4-fold) variants (137). Mandy et al. suggests this lower neutralization by Ad26.CoV2-S-vaccinated sera may be associated with mutational differences in the RBD between variants. The RBD mutations associated with immune evasion, K417T, N501Y and E484K, have been identified in the B.1.351 and P.1 variants; whereas the B.1.617 variant has acquired mutations at, L452R and T478K, associated with infectivity (137). A repetitive theme has been observed with S-based antibody responses induced by vaccines: as novel mutations are acquired by variants, the neutralization capability declines. Mechanistically, antibodies recognize epitopes on pathogens to provide immunity against a given pathogen. However, the tendency of pathogens to evolve and mutate alters these epitopes to the point where antibodies are unable to recognize their targets, leading to an unprotective humoral response. Therefore, a vaccine that induces heterologous immunity cannot only depend on spike- and RBD-specific neutralizing antibodies to provide long term and cross-variant immunity. As novel SARS-CoV-2 variants are emerging, more mutations accumulate in the S protein and the antibody responses are expected to weaken. Many studies have reported sera from Ad26.CoV2-S recipients display a substantial fold decrease or no neutralization capability against the B.1.1.529 (Omicron) variant (138–140). With over 30 mutations in the spike, antibody responses have proven to be insufficient at protecting against Omicron infections. This shifted the focus towards looking at other immune parameters conferring vaccine induced immunity against the Omicron variant. A study with macaques immunized with Ad26.CoV2-S vaccine found the presence of cross-reactive CD8^+^ T cell responses against the Omicron variant (141–143). Further evaluation of the vaccinated group found that immunologic profiles with moderate omicron-specific neutralizing antibody (NAbs) titres and negligible CD8^+^ T cell responses, and low-to-moderate Omicron NAbs titres and low CD8^+^ T cell responses fail to establish viral control in the upper respiratory tract. Meanwhile, macaques with low NAbs titres and high CD8^+^ T cell responses, and high NAbs titre and low CD8^+^ T cell response, established virological control upon SARS-CoV-2 challenge. The paper suggests that CD8^+^ T cells responses play an important role in viral protection and control against SARS-CoV-2 variants. Other studies comparing cellular responses among COVID-19 vaccines have reported Ad26.CoV2-S recipients with a sustainable CD4^+^/CD8^+^ T cell response at 8 months post-vaccination. The median CD8^+^ T cell counts for Ad26.CoV2-S recipients (0.12%) were significantly higher compared to BNT162b2 (0.016%) and mRNA-1273 recipients (0.017%) (144, 145). All in all, the waning efficacy rates of Ad26.CoV2-S vaccine against the B.1.1.529 (Omicron) variants highlighted the susceptibilities of the vaccine-induced humoral responses and emphasized the importance of cellular responses.

Evidently, Ad26.CoV2-S recipients were not protected from Omicron infections, therefore, research into homologous (same as the initial vaccine) and heterologous (different from the initial vaccine) booster vaccines were explored to re-establish the lost immunity. It was determined that both, heterologous and homologous boosting regimens were safe and effective at restoring immunity against the Omicron variant (145). A primary Ad26.CoV2-S vaccination, followed by Pfizer vaccine booster shot increased NAbs titers by 6- to 73-fold, compared to a homologous booster shot which increase titers by 4- to 20-fold (146). Spike-specific CD8^+^ T cell counts were significantly increased with the heterologous boosting regimen, whereas homologous boosting induced a more long-lasting spike-specific CD8^+^ T cell response. In South Africa, 500 000 health care workers were given a homologous Ad26.CoV2-S boosting regimen to follow and found vaccine efficacy for hospitalization increased from 63% to 84% (147). Another clinical trial among health care workers, the SWITCH Trial, compared the homologous Ad26.CoV2-S regimen with a heterologous mRNA-vaccine based regimen, and found the strongest response occurred from the mRNA-based boosting regimen (145). Heterologous mRNA-based boosters generate a higher NAb titer compared to the homologous Ad26.CoV2-S regimen (145). Additionally, all recipients of a booster shot demonstrated spike-specific IFN-γ^+^ T cell responses. Moreover, the mRNA booster (mRNA-1273: 91.7%; BNT162b2: 91.5%) has a cellular recall response compared to the Ad26.CoV2-S (72.7%) booster shot (145). Data on vaccine efficacy against infection, transmission and severe disease are not available, but are predicted to increase based on the humoral and cellular response data.

### Moderna (mRNA-1273) vaccine

The Moderna, mRNA-1273, vaccine is an mRNA lipid nanoparticle encoding the wild-type SARS-CoV-2 spike protein with a transmembrane anchor and cleavage site (148). It is an FDA-approved vaccine for all ages, consisting of a two-dose regimen, 28 days apart. Moderna (mRNA-1273) elicits a strong CD4^+^ T cell response and neutralizing antibody titre against the RBD (149). An evaluation of the mRNA vaccine-induced responses by Bezawit et al. revealed CD4^+^ T cells with cross-variant and cross-species reactivity as they recognize the B.1.1.7/B.1.351 S-protein as effectively as the wild-type SARS-CoV-2 and heterologous HCoV-HL63 S-protein (150). In addition, the antibody response had cross-reactive properties towards B.1.1.7/B.1.351 variants, leading to a robust NAb titer. The mRNA-1273-induced immunity was found effective as vaccine efficacies against B.1.1.7, B.1.351 and B.1.617 infections, after two doses of mRNA-1273, were 100%, 96.4% and 86.7%, respectively (151, 152). Moreover, mRNA-1273 recipients were protected from severe COVID-19 outcomes and hospitalizations against the B.1.1.7 (Alpha), B.1.351 (Beta), P.1 (Gamma), and B.1.617 (Delta) variants (122, 150–152). The emergence of the B.1.1.529 (Omicron) variant had negative implications for the Moderna vaccine. There was a significant reduction in effectiveness of the vaccine in protecting against infection, but protection against severe disease was still observed (153–156). Serum samples from mRNA-1273 recipients showed 41-to-84-fold reduction in neutralization ability, and vaccine efficacy declined against the B.1.1.529 (Omicron) infections to 44.0% (157, 158). With the marked reduction in protection against infection, an mRNA-1273 booster shot was encouraged. The administration of an mRNA-1273 booster restored vaccine efficacies against hospitalization and disease outcomes of COVID-19. Unfortunately, the efficacy of the booster shot against Omicron infections dropped from 71.6% to 47.4%, after 60 days (157). Research shows that the booster shots may be a temporary measure to mitigate the infectivity associated with emerging SARS-CoV-2 variants as the human population remains highly susceptible to SARS-CoV-2 infections.

The mRNA-based vaccines have proven to generate a more robust and durable memory response compared to the other COVID-19 vaccine types. The mRNA-1273 and BNT162b2, have been shown to induce spike- and RBD-specific memory B cells that cross-react with B.1.1.7 (Alpha), B.1.351 (Beta) and B.1.617 (Delta) variants and generated functional neutralizing antibodies (159). The study found that more than 50% of memory B cells cross-reacted with the three VOC (159). This cross-variant humoral response generated from the mRNA vaccines has shown to be effective as vaccine efficacy against the Alpha, Beta and Delta infections remains high (151, 152, 159). In terms of heterologous immunity, this is an ideal humoral response as it confers specificity against spike and RBD targets, establishes memory, cross-reactivity, and protects against infection. Regarding a sustainable, cross-reactive humoral response, these memory B cells increased in frequency from 3 to 6 months post-vaccination, and variant-binding memory B cells were reported to be more hypermutated compared to wild-type binding memory B cells (159). However, Choi et al. found that sera from mRNA-1273 recipients have reductions in neutralizing ability against B.1.351 (Beta), P.1(Gamma) and B.1.617 (Delta) variants, ranging from 2.1- to 8.4-fold (160). Despite the reduction in neutralizing ability, the mRNA vaccine efficacies against infections have remained intact, whereas protection against SARS-CoV-2 infections dropped with the Ad26.CoV2-S and ChAdOx1nCov-19 vaccines (75, 160). Goel et al. has associated this resilience to the early establishment of CD4^+^ T follicular cells, the presence of SARS-CoV-2-specific CD4^+^ and CD8^+^ memory T cells 3-to-6 months after vaccination, and rapid recall responses upon re-exposure of antigen (159). Notably, early CD4^+^ T follicular cells have been correlated to antibody production at 6 months post-vaccination, emphasising the role of early cellular responses in long term immunity against SARS-CoV-2. Second, the stabilization and rapid recall of SARS-CoV-2 specific CD4^+^ and CD8^+^ memory responses has been shown to correlate with long-term humoral immunity. Perhaps, as the antibody responses decline in neutralization ability, CD4^+^ T follicular cells and other T memory responses reactivate in germinal centres, and other mechanisms that facilitate memory B cells to undergo somatic hypermutation, produce functional cross-reactive antibodies that also come to play. Interestingly, T follicular cells and other populations influencing germinal centres may play an important role in adapting humoral memory responses against emerging SARS-CoV-2 variants. Moreover, inducing heterologous immunity against SARS-CoV-2 may manifest within these T cell populations that allows the adaptive immune system to repurpose their immune mechanisms to the changing landscape of SARS-CoV-2 variants.

### Pfizer/BioNTech (BNT162b2) vaccine

Pfizer/BioNTech manufactured a lipid nanoparticle mRNA vaccine that encodes full-length wild type spike protein (120). The Pfizer/BioNTech (BNT162b2) vaccine induces a high neutralizing IgG/IgA antibody titre, activates CD4^+^/CD8^+^ T cells and memory B cell responses against the S-protein, including the RBD (149, 161). A two-dose regimen of BNT162b2 has an overall 95% effectiveness at protecting against COVID-19 disease outcomes, and vaccine efficacies against B.1.1.7 (Alpha), B.1.351 (Beta), B.1.617 (Delta), and B.1.1.529 (Omicron) variants were 93.7%, 75.0%, 88.0%, and 70%, respectively (87, 124, 162, 163). Serum samples from BNT162b2 recipients showed neutralization ability declining as novel variants emerged. Antibody neutralization responses were reduced by 2.6-fold, 4.9-fold, 5.8-fold, and 41-fold against B.1.1.7 (Alpha), B.1.351 (Beta), B.1.617 (Delta), and B.1.1.529 (Omicron) (164, 165). The Pfizer vaccine has been shown to be effective until the emergence of the B.1.1.529 (Omicron) variant when the vaccine-induced immunity against infection was significantly compromised (166). Subsequently, protection against hospitalizations and disease have also been negatively impacted. Fortunately, many studies have reported that a booster dose restored the neutralization ability of recipient-sera against the B.1.1.529 (Omicron) variant (167–170). The first dose of BNT162b2 generated an early S_2_-specific IgA^+^ plasmablast response, which is an early indicator of IgA-secretion (167). Then development of an IgG^+^ memory B cell response, targeting the S_1_ subunit of the spike protein, was observed three-weeks after the first shot (167). The second dose served to boost the established humoral responses and produce functional NAbs against SARS-CoV-2 and its variants (167). Furthermore, one week after the second dose, the antibody response efficiently blocked viral entry of the B.1.1.7 (Alpha), B.1.351 (Beta), P.1 (Gamma), and B.1.617 (Delta) variants (168). However, the antibody titers and entry inhibition proficiencies were reported to decline at 3- and 6-months after the second dose (168). The BNT162b2 vaccine-induced humoral response was robust and effective in the early stages, but from one week onwards, the humoral response began to wane.

The success of mRNA vaccines against SARS-CoV-2 has been attributed to its multi-dimensional approach to priming immune mechanisms to mitigate the pathologies, infections, hospitalizations, and deaths related to COVID-19. The role of the antibody response is to neutralize extracellular viruses entering the host from the environment or prevent newly released virions from infecting additional host cells. When the antibody responses weaken, the virus can infect host cells and it is the responsibility of CD8^+^ T cells to eliminate viruses intracellularly. In addition to blocking cell attachment and entry, antibodies can also lead to killing of virus infected cells *via* ADCC (antibody dependent cellular cytotoxicity) and CDCC (complement dependent cellular cytotoxicity). The CD4^+^ T cell response orchestrates cellular mechanisms to protect against infectious agents such as SARS-CoV-2, by providing B and T cell help, generating memory, and balancing T_H_1 vs T_H_2 responses (169). The T cell immunity elicited by the BNT162b2 vaccine shows longevity and protection against SARS-CoV-2 variants (159, 171). Guerrera et al. found spike-specific CD4^+^ and CD8^+^ T memory cells, two-weeks, and 6-months after vaccination (171). These CD4^+^/CD8^+^ T memory cells were poly-functional, inducing T_H_1 cytokines (IFN-γ and IL-2), generating S-specific central memory (T_CM_) and effector memory (T_EM_) populations, and activating T stem cell memory (T_SCM_) (171). The T_SCM_ populations were stable and deemed a predictor for activated spike-specific CD4^+^ and CD8^+^ T cells at 6-months post-vaccination (171). T_SCM_ cells have self-renewal ability and function to replenish memory and effector subsets populations (172). It has been theorized that immunological memory and decade-lasting protective immunity stems from T_SCM_ populations differentiating into antigen-specific memory T cells subsets, upon re-exposure of antigen (172–175). Guerrera et al. have emphasized the stabilization and rapid recall ability of T_SCM_ populations as a signature for a sustainable cellular response (171). Another study examined memory responses associated with the Pfizer booster. They reported a strong IFN-γ^+^ or IL-2^+^ response in CD4^+^/CD8^+^ T cells, and early differentiation of SARS-CoV-2 specific CD8^+^ T cells into T_EM_ populations, which accounted for 0.02-2.92% of circulating CD8^+^ T cells (169). These CD8^+^ T cells responded to epitopes that were frequently presented by MHC alleles and conserved across SARS-CoV-2 variants, which allowed the memory T cells to develop cross-variant specificity (169). It is evident that successful COVID-19 vaccines have an intricate induction of T cell pathways and mechanisms that are diverse in their functionality and ability to recognize variants. These attributes observed in the mRNA vaccines have led to protection against B.1.1.7 (Alpha), B.1.351 (Beta), P.1 (Gamma), B.1.617 (Delta), and B.1.1.529 (Omicron) variants, despite waning antibody responses over time post-vaccination.

## COVID-19 vaccines in pipeline

The emergence of the B.1.1.529 (Omicron) variant, paralleled with the significant decline in efficacies of the 1^st^ generation COVID-19 vaccines to this variant, has mobilized attention to the future composition of COVID-19 vaccines. Currently, there are 153 vaccine candidates in clinical development, of which six candidates have shown significant protection against SARS-CoV-2 in later-phase trials (176).

(1) Covaxin (BBV152) has been developed by Bharat Biotech, in cooperation with the Indian Council of Medical Research, National Institute of Virology (176–178). It is a whole inactivated virion of SARS-CoV-2 tagged with a Toll-Like Receptor (TLR) -7/-8 agonist molecule (IMDG), and Alhydroxiquim-II adjuvant (178). In Phase III clinical trials with 25 798 participants, Covaxin had a vaccine efficacy of 77.8% against mild-to-moderate COVID-19, 93.4% against severe COVID-19, and 65.2% against the Delta variant (177, 178). This vaccine is approved for emergency use in 16 countries and currently under review for approval by Health Canada and FDA. (2) The GBP510 vaccine is developed by SK Bioscience and GlaxoSmithKline (178, 179). It is an adjuvanted self-formulating nanoparticle vaccine targeting the RBD of SARS-CoV-2. Interim Phase I/II results indicated a 100% seroconversion rate and a strong neutralizing antibody response that was 5-8 times higher than convalescent sera (178). The Phase III clinical trial results have been yet to be posted (179). (3) INO-4800 developed by Inovio Pharmaceuticals, is a prophylactic DNA vaccine targeting the SARS-CoV-2 S-protein (176, 178). The vaccine has been reported to induce a functional antibody and CD4^+^/CD8^+^ T cell response against all the VOCs, including the Delta variant (178). Phase II/II INNOVATE trial are evaluating the INO-4800 vaccine and results are pending. (4) Novavax developed a nanoparticle vaccine consisting of a stable pre-fused, coronavirus S-protein coupled with a Matrix-M adjuvant (178). In animal models, NVX-CoV2373 showed that a single-dose regimen induced high spike-specific NAbs (180). Phase III trials reported a vaccine efficacy of 90.4% against mild-to-severe disease, 100% protection against moderate-to-severe disease, and 92.6% efficacy against VOCs (181). Novavax plans to combine NVX-CoV2373 and an influenza vaccine, NanoFlu, in hopes to address SARS-CoV-2 and influenza virus in the upcoming flu season (178). A clinical study is underway for evaluating the effectiveness of combining the vaccines. (5) The SCB-2019 (CpG 1018/Alum) vaccine is a stabilized trimeric S-protein with CpG 1018 and Alum adjuvant. Developed by Clover Biopharmaceutical and Dynavax Technologies, the vaccine has shown 100% efficacy against severe disease outcomes and hospitalizations, 84% efficacy against moderate-to-severe disease and overall vaccine efficacies against the B.1.617 (Delta), P.1 (Gamma) and B.1.621 (Mu) variants of 79%, 92% and 59%, respectively (178, 182). (6) VBI Vaccines have an enveloped virus-like particle expressing S-protein with an alum adjuvant, called VBI-2902 (178). Results from Phase I/II trials reported 100% neutralizing Abs titres present in recipients, with 4.3- and 5.0-fold increases in titre after first and second shot, respectively (178, 183). With the success of VBI-2902, the company plans on testing out a tri-valent pan-coronavirus vaccine, expressing the SARS-CoV, SARS-CoV-2, and MERS-CoV spike proteins (183).

Despite the immunity acquired from natural infections of SARS-CoV-2 and vaccines, breakthrough infections emerge to produce highly transmissible and infectious variants, such as Delta and Omicron, leading to an overall decrease in immunity against SARS-CoV-2. The nature of mRNA viruses and their replicative abilities pose a difficult challenge to eliminate SARS-CoV-2 from the human population. The current COVID-19 vaccines cannot prevent breakthrough infections, and this allows the virus to circulate in humans (and other animals) and periodically lead to outbreaks. There have been three major coronavirus outbreaks in the past 20 years and inevitably the countdown towards the next major outbreak is underway^3^. By limiting vaccine efforts towards SARS-CoV-2, these reoccurring outbreaks cannot be mitigated. The idea of developing a universal (or a pan coronavirus) vaccine would ideally protect against SARS-CoV-2 and all its variants, thus preventing future coronavirus outbreaks. Morens et al. highlight the five ideal properties of a pan-coronavirus vaccine: 1) preventing SARS-CoV-2 infections and breakthrough infection, 2) inducing long-term mucosal and systemic immunity, 3) preventing community transmission, 4) establishing durable herd immunity, and 5) having universal coverage of *Betacoronaviruses* (184). To develop a pan-coronavirus vaccine, we must understand the essential protective immune mechanisms and induce these though vaccination.

## Correlation to protection

The term “correlate of protection (CoP)” is defined as a statistical measure of the level of protection associated with an immune biomarker. It is the equivalent of predicting vaccine efficacy (185). CoP establishes a threshold for protection that allows vaccine candidates to be evaluated during clinical trials and selected for approval. However, the primary reason for defining CoP is to identify the immune biomarkers that confer protection against an infectious agent (186).

Using *in vitro* neutralization assay data and observed protection from vaccine recipients and convalescent patients, Khoury et al. created a model that predicted the relationship between the neutralizing antibody response and the degree of protection elicited against SARS-CoV-2 (187). They determined that the neutralizing antibody responses elicited by vaccines and/or natural infections is an accurate predictor of protection. The model predicts when protection against SARS-CoV-2 infections will decline with time based on neutralization levels dropping, and estimates that booster shots may be required annually to maintain protection against SARS-CoV-2 infections. As mentioned earlier, neutralization capacity positively correlates with protection against SARS-CoV-2 infections (188). Moreover, the presence of spike-specific IgG antibodies correlates most accurately with neutralization. Clinically, as neutralization capacity drops, the risk of fatal outcomes increases. Dispineri et al. have outlined the absence of early Nabs to strongly correlate to mortality and delayed viral control (188). Also, it highlights the presence of NAbs being more important than the magnitude of the NAb titres, in relation to protection (188). Contrary to general belief, higher magnitude of Nabs has been inversely correlated to disease outcomes. Hospitalized patients with severe COVID-19 have a higher NAb titre compared to mild diseased and asymptomatic patients whose NAb titres are undetectable 50% of the time (189). Consistent with many papers, Trinité et al. reported the magnitude of the NAb titre significantly worsens COVID-19 disease outcomes (189–191). Lafon et al. analyzed the humoral and cellular responses between mild and severe diseased patients (192). They found C3a and C5a levels were higher in severe-to-critical diseased patients resulting in elevated anaphylaxis. Elevated NAb titre associated with severe disease leads to large numbers of antigen-antibody complexes to form and trigger the complement cascade (192). Furthermore, the study showed that CD8^+^ T cell responses were associated with low anaphylatoxin level, which were in turn correlated with milder infections (192). Many vaccine studies report the association of protection from disease outcomes to the function of cellular mechanisms. These reports suggest that cellular responses somehow prevent the exacerbation of the NAb response in mild COVID-19 disease, compared to severe COVID-19 disease. In other words, there is a protective role for cellular immunity in COVID-19 infections and disease outcomes.

Cellular immune markers are known to contribute to protection against SARS-CoV-2, but there are very few papers that statistically prove the relationship between a cellular marker and its correlation to protection. In rhesus macaques, it was observed that the depletion of CD8^+^ T cells decreased protection against re-infection of SARS-CoV-2, suggesting a role for cellular immunity in protection (193). A study on a novel SARS-CoV-2 spike protein ferritin nanoparticle (SpFN) vaccine found that an early CD4^+^ T helper cell response (expressing type 1 cytokines such as IFN-γ, TNF-α and IL-2), CD4^+^ follicular T cells expressing IL-22, and an NAb response all conferred protection against SARS-CoV-1, and SARS-CoV-2 and its variants (194). The SpFN-induced NAbs were 50% less cross-reactive against SARS-CoV-1, compared to SARS-CoV-2, however protection was maintained. Therefore, the study suggested that CD4^+^ T cell populations contributed to the protection observed against SARS-CoV-1 (194). Furthermore, looking into comparative studies of recovered COVID-19 patients with mild or severe disease states can provide insight into possible markers that contribute to disease control. Patients with mild disease had a higher proportion of SARS-CoV-2 specific CD8^+^ T cells (195). In a similar study, SARS-CoV-2 specific CD4^+^ T cells that associated with mild disease were characterized by low proliferation ability, enhanced HLA-DR expression, and limited cytokines production (196). Moreover, in mild disease patients, there was a higher proportion of M-/N-protein-specific CD8^+^ T cells compared to S-specific CD8^+^ T cells (195). This highlights the importance of non-spike T cell responses contributing to protection in mild diseases. The M- and N-proteins are conserved proteins among coronaviruses and can be a potential target for a pan-coronavirus vaccine. From convalescent COVID-19 patients, CD4^+^ T cell responses strongly correlated to anti-SARS-CoV-2 IgG and IgA titres (197). The same study reported 27%, 21% and 11% of the total SARS-CoV-2-specific CD4^+^ T cells were specific for S-, M- and N-protein, respectively; with a small percentage accounting for Nsp3, Nsp4, orf3a, and orf8. The percentage of SARS-CoV-2-specific CD8^+^ T cells included 26% for S-protein and 12% for N-protein. Immunity from natural infection reflects that vaccine approaches for SARS-CoV-2 should expand towards targeting other structural proteins, in addition to the spike protein.

## Cross-reactive responses towards SARS-CoV-2

**Figure 3:**
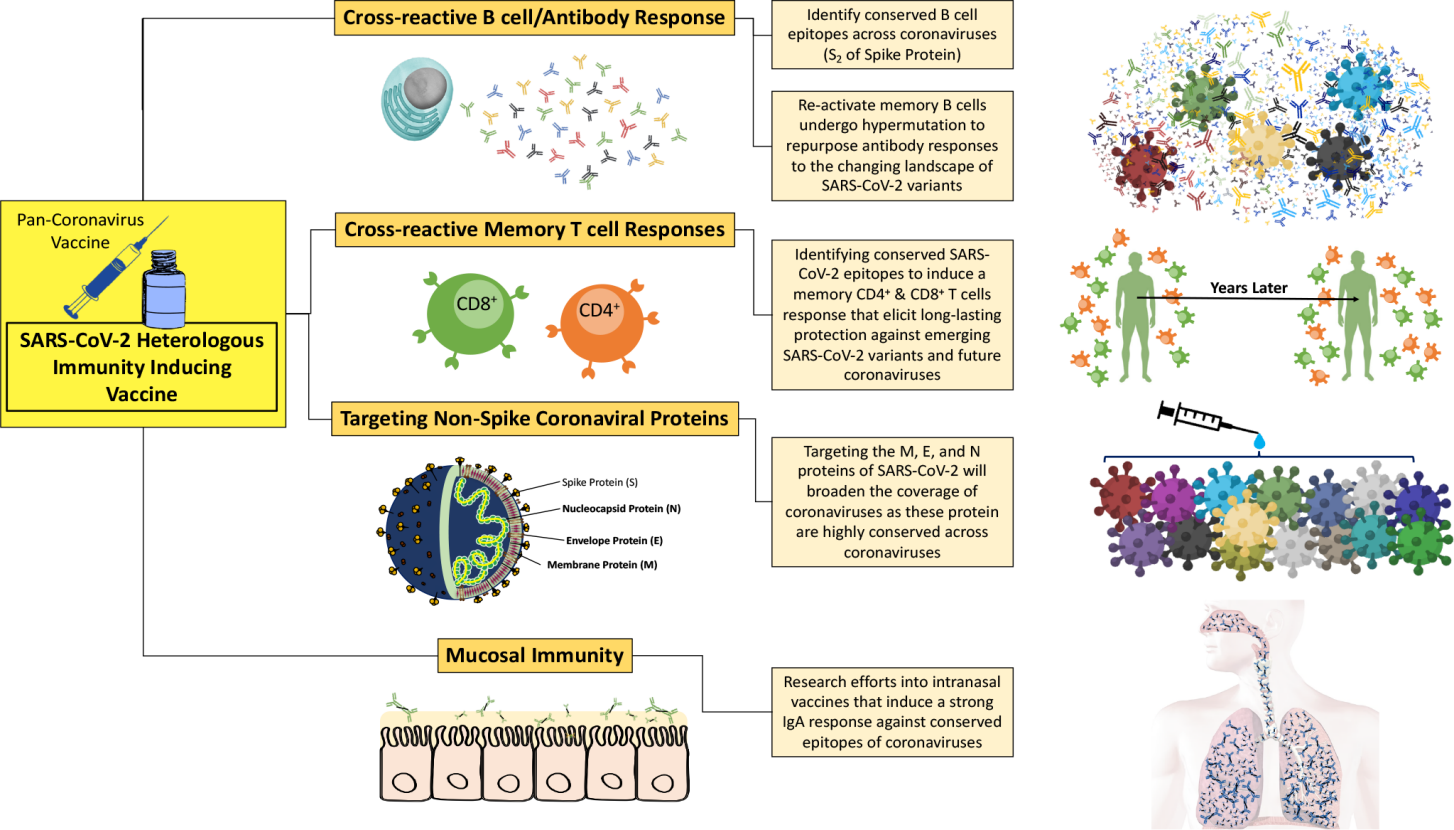
A pan-coronavirus vaccine that induces heterologous immunity across a wide landscape of coronaviruses needs to establish multiple immune parameters, including cross-reactive memory T responses, cross-reactive B cell and antibody responses, targeting of non-spike coronaviral proteins, and mucosal immunity.

Immunizations against the wild-type SARS-CoV-2 have elicited cross-reactivity towards heterologous variants of SARS-CoV-2. This includes antibody and T cell responses that cross-react with the Alpha, Beta, Gamma, Delta, and Omicron variants (**Figure 3**). Tarke et al. found that CD4^+^/CD8^+^ T cells from convalescent COVID-19 patients and vaccine recipients had similar cross-reactivity towards the Alpha, Beta, Gamma, and Epsilon variants—with a reduction in cross-reactivity among the variants of 10-22% (198). Also, amino acid sequencing analysis revealed that only 3% and 7% of the CD4^+^ and CD8^+^ T cell epitopes were affected by mutations in the VOCs, respectively. The study showed that cellular immunity can induce a cross-reactive response that is highly resistant to mutations acquired by viral variants, effectively preventing VOCs from easily escaping cellular immune system defences. Theoretically, antibody and B cell epitopes are targeted towards extracellular or surface proteins of the virus, whereas T cell epitopes have comprehensive coverage of external and internal components of the virus (198). In addition to a broadly distributed T cell epitope repertoire, HLA-restricted T cell epitopes are different from person to person, which makes viral escape difficult to achieve.

In an unexposed cohort, 40-60% of individuals had SARS-CoV-2-reactive CD4^+^ T cells, presumably induced by previous exposure to common cold coronaviruses (197). These CD4^+^ T cells have similar characteristics as the CD4^+^ T cells obtained from vaccine and natural infection induced immunity, producing IFN-γ, TNF-α and IL-2 (199). However, this population is present in higher proportions in convalescent COVID-19 patients compared to the unexposed cohort. Also, these CD4^+^/CD8^+^ T cells, found in unexposed and recovered patients, are targeting non-spike viral epitopes, including N and M epitopes which are more conserved compared to spike. This suggests that T cells targeting internal components of SARS-CoV-2 virion, which are also highly conserved proteins, can potentially lead to a cross-reactive response.

SARS-CoV-2 and human endemic coronaviruses (hCoV), such as hCoV-OC43, -HKU1, -229E, and -NL63, come from different genera within the *Coronaviridae* family. Immune responses against pre-existing hCoVs have shown to cross-react with SARS-CoV-2 which has provided insight into conserved viral proteins that can potentially be targeted for a pan-coronavirus vaccine. Finnish children and adults were compared for their antibody titres to human endemic coronaviruses to see if they cross-reacted with SARS-CoV-2 (200). The results showed pre-existing hCoV-OC43 and hCoV-229E-specific antibodies indeed cross-reacted with SARS-CoV-2 proteins. Serum samples from children had significantly higher levels of cross-reactivity to the SARS-CoV-2 S, RBD, and N proteins (p = 0.001-0.007). Not surprisingly, the highest cross-reactivity reported was to the N-protein, already known to be highly conserved among coronaviruses. A retrospective study in macaques investigated the SARS-CoV-2 specific antibody responses against SARS-CoV-1 and MERS-CoV (201). The macaques were immunized with COVID-19 vaccines, followed by natural infection with SARS-CoV-2, and serum samples were collected for analysis. They reported 123-fold and 11-fold increases in antibody binding to SARS-CoV-1 and MERS-CoV S proteins, respectively, compared to sera from pre-pandemic healthy individuals. Furthermore, S-specific antibodies from convalescent COVID-19 sera displayed an increase in binding towards hCoV-OC43, hCoV-HKU1, hCoV-229E, and hCoV-NL63 by 4.2-fold, 4.3-fold, 3.8-fold, and 1.8-fold, respectively. The paper shows the implementation of current vaccines and natural infections of SARS-CoV-2 can re-boost the cross-reactive antibody responses to effectively target SARS-CoV-1, MERS-CoV, and hCoV. The author explains that broad coverage with vaccination is feasible. Considering the decline in neutralization capacity of the vaccine induced antibody response, by 1^st^ generation COVID-19 vaccines, against the B.1.1.529 (Omicron) variants; it was unexpected that S-specific antibodies were cross-reacting to the mentioned *Alpha-* and *Beta-coronaviruses*. Further scrutiny of these cross-reactive antibodies revealed that these S-specific antibodies targeted the S_2_ subunit of spike. Other studies have reported cross-reactive antibody sera from unexposed individual to target the S_2_ subunit of SARS-CoV-2 and in some patients the S_2_ antibodies had neutralization abilities (202, 203). Natural infections of SARS-CoV-2 have been shown to induce both S_1_- and S_2_-specific antibodies, but only the S_2_-specific antibodies are able to cross-react with common cold hCoVs (203). Across coronaviruses, the S_2_ subunit is more conserved compared to S_1_, therefore the S_2_ subunit is more likely to cross-react. Taken together, identifying regions that induce cross-reactive antibody responses facilitates the effort towards developing a pan-coronavirus vaccine.

Looking at cellular cross-reactive responses, Grifoni et al. found that S-specific CD4^+^/CD8^+^ T cells are present in 60% of unexposed individuals, with SARS-CoV-2-specific CD4^+^ T cells making up most of the pre-existing cross-reactive T cells (197). Furthermore, in the unexposed cohort, memory CD4^+^ T cells had affinity for the SARS-CoV-2 N protein, and the N protein of other coronaviruses including hCoV-OC43, hCoV-229E, hCoV-NL63, or hCoV-HKU1 (197, 204). Le Bert et al. identified that memory T cells specific for N-protein from recovery patients from SARS-CoV-1, displayed a robust response against SARS-CoV-2 N protein, highlighting the cross-reactivity and long-lasting functionality of the memory subset, after 17 years (205). Moreover, unexposed individuals had SARS-CoV-2-specific T cell responses against the Nsp7 protein, which is also highly conserved among animal *Betacoronaviruses* (205). Despite the fact that the unexposed cohort demonstrated several cross-reactive memory responses, presumably derived from routine exposures from endemic human coronaviruses, a direct connection of pre-existing memory cells and improved outcomes has not been fully established.

In large part, the cellular cross-reactive responses appear to be restricted to CD4^+^ T cell subsets, and very rarely are CD8^+^ T cells subsets reported to cross-react against heterologous coronaviruses. The CD4^+^ arm of the immune system plays an orchestrating role, rather than effector or killer role, therefore they cannot directly prevent infections. Instead, they are better fit to mitigate disease severity, viral burden, and reduce prolonged disease (206). Perhaps, the lack of cross-reactive CD8^+^ T cells may be limiting cross-reactive responses from neutralizing the virus and eliciting protection against infections. Cao et al. and other studies suggest that CD8^+^ memory T cell responses should become the focus of vaccine development, to provide optimal protection against SARS-CoV-2 and other coronaviruses infections (207, 208).

Based on the idea that targeting conserved regions induces cross-reactive mechanisms, the SARS-CoV-2 M- and E- proteins may be potential targets to consider. Convalescent plasma from recovered SARS-CoV-2 patients show CD4^+^/CD8^+^ T cell epitopes are more prevalent against M- and N-protein of SARS-CoV-2 compared to the S-protein (209). This study suggests vaccine development should shift focus towards targeting more stable and conserved aspects of the SARS-CoV-2 virion, such as the M-/N-protein, rather than the rapidly mutating S-protein. Recently, a study classified immunodominant T cell epitopes from patients that recovered from an acute infection of COVID-19. They found 135 epitopes, 15-mer peptides covering the envelope, membrane, and nucleocapsid proteins (210). There were 10 N-, M- and E- peptides that showed high affinity binding to human leukocyte antigen (HLA) class II, using *in vitro* HLA binding assays. Of these, three peptides triggered CD4^+^T cell responses in more than 55% of patients. Two of the three immunodominant peptides are from the M-protein, named Mem_P30 (aa146-160) and Mem_P36 (aa176-190), and one peptide is from the N-protein, Ncl_P18 (aa86-100). The identified peptides had high affinity binding to HLA-DRB1^*^11:01 and induced a T cell derived IFN-γ response (210). In addition, Mem_P30 and Mem_P36 were capable of binding to more than 12 HLA-molecules with an affinity of 1000nM or better. The characterization of the T cell responses and their targeted epitopes in recovered COVID-19 patients, shows a potential multi-targeted approach of vaccines directed against M, N and E structural proteins. Additionally, the paper discusses the importance of classifying epitope repertoires, as it provides a map of viral peptides that our immune system is responding to, which can be implemented in vaccine design.

To understand the landscape of SARS-CoV-2 immunodominant epitopes, we also need to consider epitopes derived from non-canonical ORFs. Due to the promiscuous nature of the SARS-CoV-2 RNA replication machinery, the virus produces alternative or out-of-frame ORFs, which can contribute to the epitope repertoire that vaccines can target. Weingarten-Gabbay et al. identified nine viral peptides originating from out-of-frame ORFs, from S and N coding regions, that were expressed on several HLA-I molecules (211). Also, six of the viral peptides derived from out-of-frame ORFs were predicted to bind HLA-A^*^02:01, which is a well-distributed HLA molecule across the human population and important in antigen presentation to T cells (212). Interestingly, these viral peptides were immunogenic in transgenic HLA-A2 mice, inducing a strong CD8^+^ T cell and IFN-γ response. From the study, it was found that 25% of the HLA-I peptides detected were derived from out-of-frame ORFs from S and N coding regions, highlighting the ability of the immune system to target viral epitopes derived from non-canonical ORFs as well as canonical ORFs. Weingarten-Gabbay et al. has introduced the idea that viral epitopes from non-canonical ORFs can serves as vaccine targets. However, additional studies need to be conducted on the immune parameters it induces, the degree of cross-reactivity and protection against infection elicited by these viral epitopes derived from non-canonical ORFs.

## Conclusion

Finding the immune markers and mechanisms that confer or contribute to protection is essential in developing a pan-coronavirus vaccine. The current COVID-19 vaccines are inducing heterologous immunity, and the immunity is cross-reactive and cross-protective to a certain extent against SARS-CoV-2 VOCs. By analyzing the vaccine induced immunity from 1^st^ generation vaccines, it is evident that antibody responses are effective at protecting against the wild-type SARS-CoV-2 and its variants. However, as SARS-CoV-2 variants accumulated mutations in the spike protein, sera from vaccine recipients declined in neutralization capabilities where protection against infection was reduced. This raised the question about the reliance of antibody responses and their ability to effectively protect the human population against SARS-CoV-2 and its variants. Secondly, it encouraged research to identify cellular immune responses that may contribute to coronavirus protection and pinpoint cross-reactive mechanisms that broaden coronavirus immunity outside of SARS-CoV-2. The correlation of protection is clearly outlined for the humoral response in terms of NAb titre levels, neutralization capacity, and the onset of the NAbs response. In contrast, cellular responses are ill-defined when it comes to protection. However, repeated examples of CD4^+^/CD8^+^ T cells associated with protection against severe disease have provided some insights. Additionally, cross-reactive mechanisms identified from unexposed individuals have allowed researchers to identify conserved epitopes that induce cross-reactive immune responses and encouraged targeting viral proteins other than the spike protein. Recognition of cross-reactive immune responses have contributed to broadening the protective coverage toward other coronaviruses and made the idea of developing a universal vaccine for coronaviruses more feasible. Additionally, epitope studies and databases like IEDB assist in vaccine design and allow the targeting of selected epitopes that can produce heterologous immunity that is cross-reactive, comprehensive in coverage of SARS-CoV-2 viral proteins and other coronaviruses infections, and inducive of a long-lasting, multifaceted humoral and cellular responses.

## Author contributions

RP and BA conceptualized the article and collected the literature. RP wrote the first draft. BA and RP revised the manuscript. All authors contributed to the article and approved the submitted version.

## Funding

This work is funded by project grants (PS165854 and PS173314) from the Canadian Institutes of Health Research (CIHR) to BA.

## Acknowledgments

We thank Dr. M.S. Peppler for reading, editing and providing critical comments.

## Conflict of interest

The authors declare that the research was conducted in the absence of any commercial or financial relationships that could be construed as a potential conflict of interest.

## Publisher’s note

All claims expressed in this article are solely those of the authors and do not necessarily represent those of their affiliated organizations, or those of the publisher, the editors and the reviewers. Any product that may be evaluated in this article, or claim that may be made by its manufacturer, is not guaranteed or endorsed by the publisher.
